# Exploring Young People’s Views on Pharmaceutical Care for Long-Term Illnesses in Primary Care Settings

**DOI:** 10.3390/healthcare13212796

**Published:** 2025-11-04

**Authors:** Mohammed Almunef, Julie Mason, Chris E. Curtis, Zahraa Jalal

**Affiliations:** 1 Department of Pharmacy Practice, College of Pharmacy, Qassim University, Buraydah 51452, Saudi Arabia; 2 School of Pharmacy, College of Medicine and Health, University of Birmingham, Edgbaston, Birmingham B15 2TT, UK; j.mason@bham.ac.uk (J.M.); c.e.curtis@bham.ac.uk (C.E.C.); z.jalal@bham.ac.uk (Z.J.)

**Keywords:** pharmacist, young people, young adult, chronic disease, questionnaire, cross-sectional study

## Abstract

**Background/Objectives**: According to recent literature, the prevalence and incidence of long-term illnesses such as asthma and diabetes in young people have substantially risen over the past 13 years. Recent figures indicate that, in England, 4.10% of all prescriptions were prescribed for young people. The aim of this study was to investigate young people’s perspectives of pharmaceutical services provided by primary care pharmacists relating to medication. **Methods**: A cross-sectional survey using both online and paper-based tools was conducted from March to November 2019. The population for this survey was young people from 18 to 24 years old registered as students at one of the universities in England. The survey consisted of twenty-four questions, and they were a mix of closed-ended questions, such as multiple-choice and Likert scales, and open-ended questions. **Results**: A total of 210 out of 800 survey responses were completed from different recruitment sources, achieving a response rate of 26.25%. Most participants were female (62.38%), and the most frequent age was 18 years (35.24%). Among participants, 15.70% were diagnosed with long-term illnesses, of which 33.33% were reported as the respiratory disease, asthma. Pharmacists were not utilised as a source of information for young people, with the majority (60.60%) obtaining information from their doctors. Most of the participants (96.97%) had not taken part in a Medicines Use Review (MUR) or New Medicine Service (NMS), and 78.79% were not aware of any services or support groups by their pharmacist. Among different healthcare professionals, GPs and hospital doctors were the most frequently reported to discuss with young people about their illnesses. **Conclusions**: There is an opportunity to further develop pharmaceutical services and support by primary care pharmacists for young people with long-term illnesses. Policymakers and primary care pharmacists in the future could utilise the perceptions and opinions of young people found in the current study to inform the development of primary care pharmacy services to meet young people’s needs and perceptions. These results are of benefit to policymakers in assisting in the development of pharmacy services. Further research will enhance understanding of the perceptions of young people about the pharmaceutical services offered by primary care pharmacists with respect to medications.

## 1. Introduction

Young people are often perceived as generally healthy, but those living with long-term illnesses face significant challenges in managing their health. These challenges include navigating complex treatment regimens, maintaining adherence, and balancing healthcare responsibilities with educational and social demands. Poor adherence in particular has been reported as more prevalent among younger populations compared with adults, leading to avoidable complications and reduced quality of life [1]. For the purposes of this study, ‘young people’ are defined as individuals aged 10–24 years, in line with definitions provided by the World Health Organisation [2]. This age range was chosen to capture the transition from adolescence to early adulthood, which is a critical period for managing long-term health conditions. Pharmacists are well positioned to support patients with long-term conditions through accessible advice, medicines management, and structured services such as the New Medicine Service (NMS) and Medicines Use Reviews (MURs). However, the majority of pharmacy services in England and internationally have traditionally been directed towards older adults, leaving younger people with fewer opportunities for meaningful engagement with pharmacy support [3]. This is despite evidence showing that early and targeted interventions can improve adherence and reduce the long-term burden of chronic illness in this age group.

The prevalence and incidence of long-term illnesses such as asthma and diabetes in young people has risen substantially over the past 13 years [4]. Recent figures indicate that, in England, 4.10% of all prescriptions were prescribed for young people. More than 45 million prescriptions were dispensed for young people in 2017 by pharmacists [5]. Young people represent a vulnerable population with distinct healthcare needs that make them a critical target for pharmaceutical care in primary settings. Young people are frequently affected by chronic illnesses like asthma, diabetes, and mental health issues from an early age, making continuous and effective medication use essential to avoid serious long-term health consequences [2]. Moreover, this age group often demonstrates low adherence to prescribed therapies, particularly in conditions such as ADHD and depression, due to developmental, social, and psychological factors [6]. Additionally, young people are in the process of developing autonomy over their health decisions, yet often lack the necessary health literacy to engage effectively with medication regimens [7]. Young people who are taking medications regularly are at high risk of facing different issues related to medicine taking. These issues include medication non-adherence and improper administration.

Emerging studies also suggest that young people underutilise pharmacists as a source of medicines information, relying more heavily on doctors or online resources [8]. Barriers include lack of awareness of available services, limited pharmacist engagement with younger populations, and practical challenges such as privacy concerns [9]. These factors may contribute to the observed lower levels of adherence and reduced uptake of pharmaceutical services in this group. As medicines experts, pharmacists could potentially improve young people’s knowledge regarding effective use of medication through the provision of pharmaceutical services [10]. Pharmacists within primary care settings are well positioned to provide tailored counselling, promote medication adherence, and serve as accessible points of intervention for health promotion [11]. Therefore, implementing pharmaceutical care strategies for young people in primary care is not only clinically essential but also an opportunity to shape lifelong positive health behaviours and reduce long-term healthcare costs.

Pharmaceutical care has been defined in the recent literature as a pharmacist-led, patient-centred approach that aims to prevent drug-related problems and optimise medicine use while being monitored by evidence-based quality measures. As a crucial, supplementary aspect of larger health system care, it differs from general healthcare in its disciplined focus on medication therapy as the tool and the goal for improving outcomes [12]. The majority of studies investigating pharmaceutical services in primary care settings focus on either pharmacists’ views or adult patient populations. There is a paucity of published literature regarding young people’s perspectives of the pharmaceutical services provided by primary care pharmacists, i.e., general practice (GP) and community-based pharmacists, relating to medication [10].

Despite these concerns, there is limited research exploring young people’s perceptions of pharmacy services and how these services might be tailored to their needs. Understanding these perspectives is essential to informing the future development of pharmacy services that are more responsive and effective in supporting young people living with long-term illnesses. In light of this gap, the present study aims to explore the perceptions of young people aged 18 to 24 years with long-term conditions regarding pharmaceutical services provided by primary care pharmacists.

The secondary aims of this study were as follows:

- To investigate the presence and history of long-term illnesses among participants.
- To determine the perceived knowledge of young people about their illness.
- To identify the preferred way for young people to find information about their illness.
- To determine the impact of long-term illness on young people’s life.
- To explore medication use experiences for young people with long-term illnesses
- To investigate current pharmacy services provided to young people with long-term illnesses.
- To explore the perceptions and experiences of young people with long-term illnesses on the provision of pharmacy services.

## 2. Materials and Methods

### 2.1. Study Design

The survey in this study was designed after identifying and considering issues relevant to addressing the primary and secondary aims listed above. Survey content was informed by utilising the gaps in the published literature that had not been addressed by previous studies [13].

The survey covered the six following themes:

○ The perceived knowledge of young people about their illness.
○ The preferred way for young people to find information about their illness.
○ The impact of long-term illness on young people’s life.
○ Medication use experiences for young people with long-term illnesses
○ The current pharmacy services provided to young people with long-term illnesses.
○ The perceptions and experiences of young people with long-term illnesses on the provision of pharmacy services.

The survey consisted of twenty-four questions uploaded on an online platform, “Online surveys”, to facilitate distribution and respondent completion. The questions were a mix of closed-ended questions, such as multiple choice and Likert scale, and open-ended questions. Responses were recorded on a 5-point Likert scale, where 1 represented the least positive response (e.g., strongly disagree/poor) and 5 represented the most positive response (e.g., strongly agree/excellent). The diversity in question types allowed focused and detailed answers to be obtained. The identified subjects were classified into themes, and questions were structured to address these points. Inclusion criteria in this study: young people aged 18 to 24 years. Participants who reported not having a long-term condition were screened out at the first question and did not proceed further with the survey. Only those confirming a long-term condition continued to complete the remaining items.

An agile approach was followed, where end users were involved in the design of the survey. Then it was validated by the research team and end users (young people at university level). Before distributing the final survey, a pilot test was conducted with a group of ten pharmacy students from an academic institution in England to assess the clarity, relevance, and structure of the questionnaire. The pilot participants were asked to review the questions and provide feedback on wording, flow, and comprehensibility. Based on their input, several modifications were made to enhance the survey’s validity and reliability. For example, ambiguous terms such as “medication experience” were rephrased to “your experience with taking prescribed medicines,” and the Likert scale format was revised for consistency across all sections. These refinements contributed to ensuring that the survey was clearly understood and interpreted as intended by the target population.

### 2.2. Survey Population

The population for this survey was young people from ages 18 to 24 years registered as students at an academic institution in England. Considering the ethical sensitivities of asking young people about long-term illness—such as the potential for emotional discomfort, recalling difficult experiences, concerns over confidentiality, and possible stigma—we limited recruitment to students from the same institution as the researchers. This approach ensured that participants had direct access to established welfare and counselling services should they require support during or after the survey. Students from different year groups and schools were surveyed for this study.

However, sample size calculation was not conducted as the survey was distributed by convenience sampling. In this study, we employed a convenience sampling method, which involved recruiting participants who were readily accessible and willing to participate. This approach was selected due to its practical feasibility.

### 2.3. Ethical Considerations

This research gained ethical approval from the respective academic institution’s Ethics Committee (ERN_17-1672). Respondents were informed that participation was entirely voluntary, all the information collected in the course of the research would be kept strictly confidential, and all responses would be anonymised. A Participant Information Sheet was provided to explain how the research would be conducted and the details for participation. Moreover, the right to withdraw from the survey at any time was assured to the participants without the need to provide any explanation. Participants were informed they could withdraw at any point prior to submitting the questionnaire. As the survey was anonymous, it was not possible to withdraw responses after submission.

### 2.4. Data Collection

Data collection was conducted from March to November 2019. The survey was distributed via two formats: online and paper. The online version of the survey was sent to a sample of students at the University of Birmingham via e-mail in March 2019. The survey invitation was distributed via the university’s student administration office, which sent the email directly to all eligible students. Researchers did not have access to individual student email addresses, thereby maintaining confidentiality and anonymity. Undergraduate programme managers in pharmacy, medicine, nursing, biomedical sciences and dentistry schools were contacted to facilitate the distribution to approximately 5000 students. Additionally, an invitation for participation was posted on a teaching platform page for pharmacy students. Four reminder emails were sent via programme managers through the allocated period of data collection. These reminders were sent in April, May, September, and October 2019. The survey was distributed electronically and configured to allow only one response per participant login, preventing duplicate submissions even though reminders were sent over several weeks. To ensure the opinions and past experiences of students who were unable to participate online, a paper format of the survey was distributed to different groups of students in different schools at the university. Many on site, campus locations were also targeted for face-to-face recruitment. The survey took approximately 10–15 min to complete. Paper questionnaires were collected anonymously, with no identifying information recorded. Completed questionnaires were placed by participants directly into a sealed collection box to ensure confidentiality. These were then transcribed into the secure study database by the research team, and no attempt was made to identify respondents.

### 2.5. Data Management

Survey responses were coded by assigning codes from 1 to 5 for Likert scale questions. For Yes, No, and Not sure answers, the codes 1, 2, and 3 were used. Multiple choice questions had different coding based on the available choices, starting from 1 to 4 to 1–9. After coding, data was entered into the Statistical Package for the Social Sciences (SPSS) v26 (IBM Corp. in Armonk, NY, USA). Finally, all entered data was reviewed and checked by the main researcher to prevent any errors throughout the process. This was performed via screening and matching the answers and their equivalent codes.

### 2.6. Data Analysis

Data was analysed using SPSS v26 (IBM Corp. in Armonk, NY, USA) and Microsoft Excel. Descriptive analysis was performed on the data mainly by using SPSS, and for some questions Microsoft Excel was used. Analysis of the free-text answers to open-ended questions was conducted by categorising answers and then quantifying them.

In this study, diseases were classified according to World Health Organisation (WHO) ICD-11 classification of disease [14]. Participants were given a list of diseases to choose from. Eczema, atopic dermatitis, skin allergy, and oral allergy syndrome fell under skin diseases; asthma fell under diseases of the respiratory system; chronic pancreatitis, irritable bowel syndrome (IBS), and ulcerative colitis fell under diseases of the digestive system; depression and anxiety fell under mental health; diabetes, lactose intolerance, polycystic ovary syndrome (PCOS), and hypothyroidism fell under endocrine; aortic valve stenosis fell under cardiovascular; and anaphylaxis fell under allergy. Other illnesses that were classified as (other) included anaemia (diseases of the blood or blood-forming organs); brain tumour (diseases of the central nervous system); endometriosis (diseases of the female genital system); systemic onset of juvenile idiopathic arthritis (diseases of the musculoskeletal system or connective tissue); and ptosis (diseases of the visual system).

## 3. Results

A total of 210 out of 800 survey responses were completed from different recruitment sources, achieving a response rate of 26.25%. All the responses to the online and paper surveys met the inclusion criteria.

### 3.1. Demographic Details for the Respondents

Of 210 responses, most were completed by females, 62.38% (131/210), followed by males, 36.67% (77/210), and prefer not to say, 0.95% (2/210). Participants’ age range was between 18 and 24 years old. The most frequent age was 18 years old, 35.24% (74/210). Among participants, 15.70% (33/210) were diagnosed with long-term illnesses. The majority of participants with long-term disease, 33.33% (11/33), had respiratory disease, all of which was reported as asthma, followed by endocrine diseases (7/33), 21.21%. The latter included diabetes 12.12% (4/33), lactose intolerance 03.03% (1/33), polycystic ovary syndrome (PCOS) 03.03% (1/33), and hypothyroidism 03.03% (1/33). Although six participants were diagnosed with more than one long-term illness, the majority were diagnosed with only one chronic illness. Table 1 shows the demographic characteristics of the young people involved in the study and the prevalence of different long-term illnesses reported.

### 3.2. The Relationship Between Participants’ Age (In Years) and the Presence of Long-Term Illness

The presence of long-term illness was most frequent at the age of 18, followed by 20. Table 2 below outlines the relationship between participant’s age (in years) and the presence of long-term illness.

### 3.3. The Relationship Between Participants’ Gender and the Duration of Long-Term Illness

Among the respondents, nine out of 33 (27.27%) were diagnosed with long term illness for more than 15 years. Around a third of the participants (33.33%) were diagnosed with long term illness for less than 5 years. Table 3 presents the relationship between participants’ gender and the duration of long-term illness.

### 3.4. Determination of the Perceived Knowledge of Young People About Their Illness

**Figure 1:**
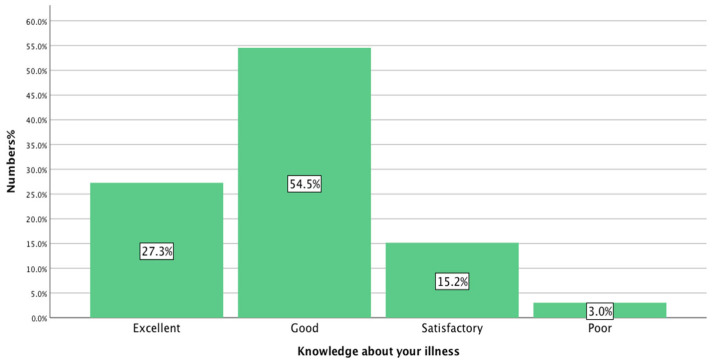
Determination of the perceived knowledge of young people about their illness (n = 33).

A small majority of respondents, 54.54% (18/33), answered that they have ‘good knowledge’ about their illness, while responses of ‘excellent’, 27.27% (9/33), or at ‘least satisfactory’, 15.15% (5/33), were also recorded. However, 3% of the respondents reported that they had poor knowledge regarding their illness, as shown in Figure 1.

### 3.5. Age-Appropriate Information

When asked, “In your opinion, do you feel there is enough age appropriate information made available to support you making decisions regarding your condition?” 42.42% (14/33) answered “Yes”, 36.36% (12/33) answered “No”, 21.21% (7/33) answered “Not sure”.

### 3.6. The Preferred Way for Young People to Find Information About Their Illness

**Figure 2:**
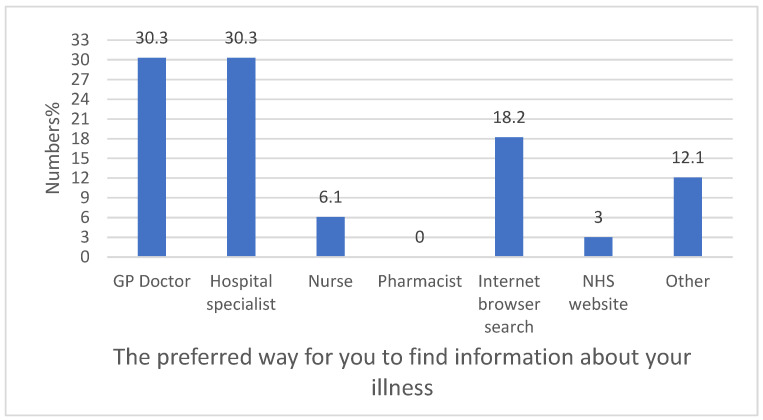
The preferred way for young people to find information about their illness (n = 33).

It can be seen from the data in Figure 2 that pharmacists were not utilised as a source of information for young people, whereas the majority obtained information from their doctors.

### 3.7. Using Digital Media for Educational Purposes or Reminder Services

The majority of the participants, 72.72% (24/33), agreed with the principle of using digital media for educational purposes or reminder services. Only 12.12% (4/33) disagreed with this idea, and 15.15% (5/33) neither agreed nor disagreed.

### 3.8. Determining the Impact of Long-Term Illness on Young People’s Life

#### 3.8.1. The Impact of Long-Term Illness on Young People’s Social Life

The following questions were used to evaluate the impact of the long-term illness on young people’s social life:

“How often do you get symptoms of your illness?

How would you rate the effect of your illness on your relationships with other people your age?”

**Figure 3:**
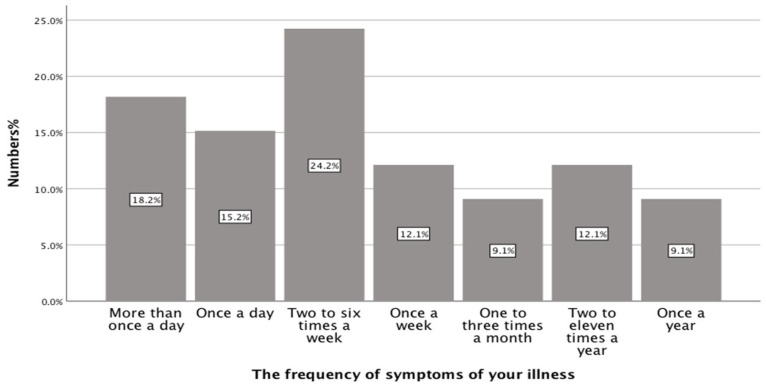
The frequency of symptoms among participants (n = 33).

Approximately one third of the participants (33.33%) experienced symptoms of their illness once a day or more frequently, while 36.36% (12/33) experienced symptoms at least once a week. The remainder of participants experienced symptoms of their illness less than once a week, as can be seen from Figure 3 (below).

51.51% (17/33) of young people rated the effect of their illness on their relationships with other people their age as “low” to “moderate”, and 30.30% (10/33) answered “none”. However, 15.15% (5/33) and 3.03% (1/33) rated “high” and “very high” to describe the effect of their illnesses on their relationships.

#### 3.8.2. The Impact of Long-Term Illness on Young People’s Education and Learning

To investigate the effect of long-term illnesses on educational development, three questions were asked:

“How would you rate the impact of your illness on your education and learning?

How many school/college/university days per year on average have you missed because of your illness?

In the last year, how often, if at all, do you actually attend hospital or GP visits for your illness?”.

A large portion of the respondents rated the impact of their illness on their education and learning as “low”. 21.20% (7/33) answered “moderate”, and 12.10% (4/33) answered “none” for this question. In contrast, 12.10% (4/33) rated the impact as “high” and 3% (1/33) as “very high”.

**Figure 4:**
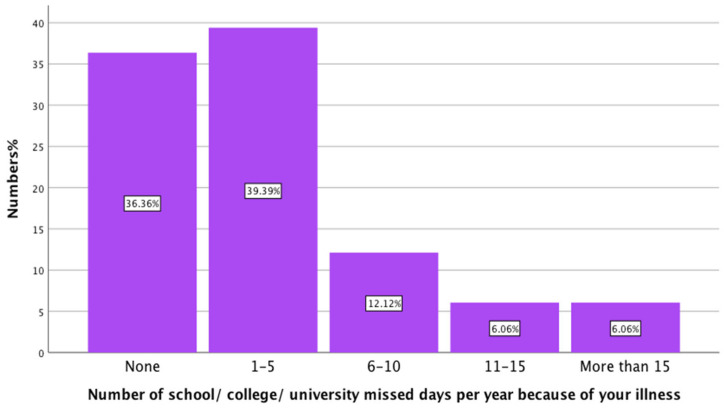
The effect long-term illness on YP attendance to university (n = 33).

When asked about the days that young people missed due to their illness, most of the participants, 63.63% (21/33), missed at least one day of university per year because of their illness. While the rest, 36.36% (12/33) of the participants, did not miss any university days because of their illness. Figure 4 presents the effect of long-term illness on YP attendance at university.

To the question about attending hospital or GP visits for YP illness, 42.40% (14/33) of respondents answered “sometimes”, 30.30% (10/33) answered “rarely”, 12.10% (4/33) answered “never”, 9.10% (3/33) answered “very often”, and 6.10% (2/33) answered “all of the time”.

### 3.9. Exploring Medication Use Experiences for Young People with Long-Term Illnesses

#### 3.9.1. The Number of Prescribed Medicines

It was of note that, 13/33 (39.40%) of the participants were not taking any medicines to manage their illness. While 10/33 (30.30%) were taking one medicine, 5/33 (15.20%) were taking two medicines, and 4/33 (12.10%) of them were taking three medicines to manage their illness. One participant (3%) was prescribed five medicines to manage their illness.

#### 3.9.2. The Frequency of Medicine Administration

**Figure 5:**
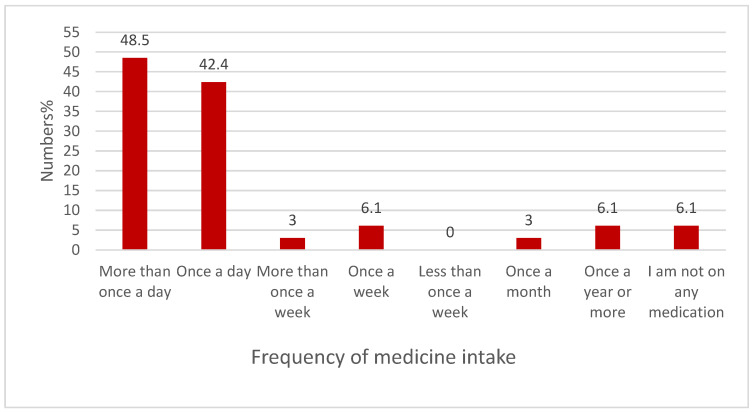
The frequency of medicine administration (n = 33).

Figure 5 below illustrates the frequency of medicine administration among participants. Almost half of the respondents, 48.50% (16/33), chose “more than once a day”, followed by “once a day”, 42.40% (14/33).

#### 3.9.3. Prescription Collection

The majority of young people (78.79%) reported that they collect their medicine by themselves. Their parents collect the prescriptions for 12.12% (4/33) of YP, and others, such as a partner or friend, for 9.09% (3/33) of the sample.

#### 3.9.4. Medication Adherence

**Figure 6:**
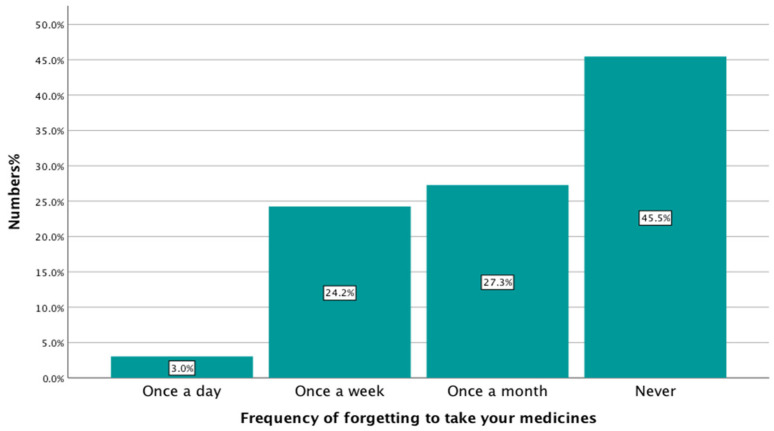
Medication adherence among the respondents (n = 33).

A higher percentage of young people reported that they forgot to take their medication at least once a month, 54.50% (18/33), than those that never forgot, 45.50% (15/33), as shown in Figure 6.

### 3.10. Current Pharmacy Services Provided to Young People with Long-Term Illnesses

#### 3.10.1. Medication Counselling

When asked if the participants had been counselled by their pharmacists, 42.42% (14/33) answered “never”, 21.21% (7/33) answered “rarely”, 6.06% (2/33) answered “sometimes”, 12.12% (4/33) answered “usually”, 9.09% (3/33) answered “always”, and 9.09% (3/33) answered “not sure”.

#### 3.10.2. New Medicines Service (NMS) and Medicines Use Reviews (MUR)

Most of the YP who took part in this study, 96.97% (32/33), had not taken part in an MUR or NMS. Only 3.03% (1/33) had participated in these services.

#### 3.10.3. Informing Young People About Pharmaceutical Services and Health Information Sources

A total of 78.79% (26/33) of YP had never heard about any services or support groups by their pharmacist, and 15.15% (5/33) of them were not sure. 6.06% (2/33) had been told by their pharmacist about services or support groups. Moreover, only 18.18% (6/33) had been told by their pharmacist about an online information source, while 72.73% (24/33) of YP had not been told about any information source by their pharmacist, and 9.09% (3/33) of them were not sure.

### 3.11. Experience of Young People with Healthcare Professionals

**Figure 7:**
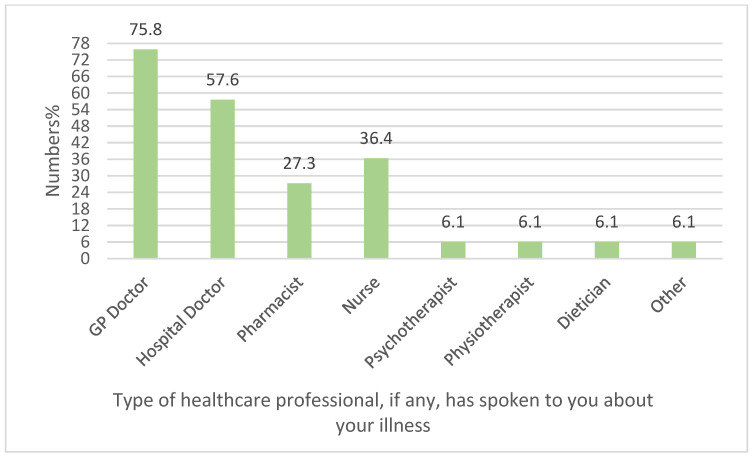
Experience of young people with healthcare professionals (n = 33).

From the graph below (Figure 7), we can see that when asked about the type of healthcare professional, if any, that had spoken to YP about their illness since turning 18 years of age, 27.27% (9/33) of YP selected pharmacist. Among different healthcare professionals, GPs and hospital doctors were the most frequently reported as those who had spoken to YP about their illnesses, 75.75% (25/33) and 57.57% (19/33), respectively. Nurses were the third most frequently selected answer by 36.36% (12/33) of the participants. Psychotherapist, physiotherapist, and dietician were selected by 6.06% (2/33) of the participants for each profession. Interestingly, 6.06% (2/33) selected ‘other’ as an answer and explained their selection further by mentioning that none of the healthcare professionals had spoken to them about their illness.

### 3.12. What Young People with Long-Term Illnesses Need from Pharmacists

#### 3.12.1. Pharmacist Assistance in the Management of Long-Term Illnesses in Young People

Young people were asked, “How do you think a Pharmacist could help you manage your illness?” and could select more than one response. The most frequent answer selected by the participants was “a phone call from the pharmacist to see how I am getting on”, 51.51% (17/33). Some of the young people, 30.30% (10/33), expressed a desire to have “a one-to-one meeting with a pharmacist when I collect my medicines”, and 21,21% (7/33) preferred “send me reminders to take my medicine”. 9.09% (3/33) selected “visit my school/college/university to provide education about medicines”.

#### 3.12.2. The Most Required Information Needs from a Pharmacist

When given a choice of options, almost three quarters of young people with long-term illnesses expressed the need for information about side effects from pharmacists, 72.72% (24/33), 45.45% (15/33) answered “the ways of taking medication”, 39.39% (13/33) answered “dose”, 36.36% (12/33) answered “the indication”, and 12.12% (4/33) answered other information needs, for example, medication storage and the complications of not taking the medicines (i.e., non-adherence). In this question, the participants were allowed to select more than one answer.

## 4. Discussion

The prevalence of long-term illness among participants in this study was 15.71%, and this figure is almost in line with national statistics of 17% [15]. The number of individuals having a history of long-term illnesses for more than 15 years was a reflection that asthma was the most common condition, 33% (11/33) reported, which was likely to have been diagnosed in childhood, followed by diabetes, 12.12% (4/33). Almost half of participants (54.50%) felt that they had good knowledge about their illness, suggesting that this good level of knowledge surrounding long-term illnesses—as felt—could have an impact on medication adherence. Additionally, this could have an effect on seeking further advice from a pharmacist, as they were not likely to do so in this study.

It was notable in the present study that the preferred way for young people to find information about their illness was from their doctor. The majority (60.60%) of young people found information from GP doctors and hospital specialists. Also, some participants (18.20%) preferred the use of internet browser searches as sources of information about their illness. This is predictable, as technology has become an essential part of a young person’s life [16]. Importantly, pharmacists were not utilised at all as a source of medicine information by the participants. An explanation for this could be that around a third of the sample had not needed to take medication frequently to manage their condition. The relatively low reported use of pharmacists among our respondents may be partly explained by the age-related prevalence of long-term conditions. Conditions such as asthma, type 1 diabetes, and mental health disorders are more common in younger populations, while others—such as hypertension and type 2 diabetes—are typically diagnosed later in life. This difference in disease profile could contribute to the limited pharmacist engagement observed in our sample, as younger individuals may have fewer medication-related needs requiring ongoing pharmaceutical care.

In the present study, almost half of young people forgot to take their medication at least once a month, 54.50% (18/33). This is an indication of low medication adherence among this population, which corresponds to conclusions found in the previous literature [9]. However, the adherence rate observed here appears slightly lower than that reported in some earlier England-based studies on young people with long-term illnesses, where regular follow-up and structured pharmacist involvement were more common. This difference may be due to the complete absence of pharmacist engagement in our sample, limited access to medicine-use services such as NMS or MUR, or the relatively lower severity of illness in some participants compared to clinical populations in hospital-based research.

Moreover, non-adherence in young people has previously been reported as being higher than in adults [17,18]. The reasons for non-adherence in this study may include a combination of factors: a perception among some participants that their illness was manageable without strict adherence; forgetfulness and competing priorities in daily life; limited awareness of adherence-support tools; and a lack of engagement with pharmacists, who could otherwise provide personalised counselling. Barriers specific to youth—such as concerns about stigma, a desire for independence from parental oversight, and uncertainty about how to approach healthcare professionals—may also play a role. To investigate the impact of a long-term illness on young people’s social lives, participants were asked if their long-term illness had affected them socially; 69.70% of them indicated such an impact. In addition, when asked about the impact of a long-term illness on their education and learning, most of the respondents (87.90%) identified that their long-term illness had an impact on educational and learning development. These impacts were reported in previous published literature [19]. The low medication adherence level reported in this study could provide reasoning for these impacts on social and educational development for young people.

When asked about medication counselling and if the participants had been counselled by their pharmacists, 42.40% (n = 14) answered “never” and 21.20% (n = 7) answered “rarely”. This indicates that a small number of counselling sessions were conducted by primary care pharmacists with young people with long-term illnesses. Almost all the participants, 96.97% (32/33), had not taken part in an MUR or NMS. It can be concluded that young people lack interaction with pharmaceutical services and pharmacists. This finding is consistent with earlier research showing that young people are among the least frequent users of structured pharmacy services, possibly reflecting both a lack of awareness among patients and limited targeting of this age group by service providers. The finding that almost all participants had not engaged in MURs or NMS may partly be explained by the nature of these services. Both required informed consent and a level of capacity to understand and benefit from the intervention, which may have made pharmacists hesitant to offer them to younger patients. Additionally, since MURs are no longer part of NHS service provision, it is important to interpret these results in the historical context of when the data were collected, rather than in light of current practice. On the other hand, it might also suggest that either pharmaceutical services might not be available or that the services are there but are not being used. There is an opportunity for the provision of pharmaceutical services and support by primary care pharmacists to young people with long-term illnesses, but sometimes this opportunity is missed. Previous evidence shows that this could be due to a lack of confidence when dealing with young people or the unwillingness of primary care pharmacists to take on more responsibilities [20]. Shortage of training and support for pharmacists could be considered among the contributing factors [20]. On a national level, it is recognised that there is a gap in the transition of healthcare between childhood and adulthood [21]. Providing pharmaceutical services that specifically target adherence issues, for instance, NMS and, more recently, enhanced structured medicine use reviews by clinical pharmacists working within primary care networks could be valuable opportunities for pharmacists to engage further with young people with long term illnesses.

### 4.1. Strengths and Limitations

A major strength of this survey is that few published studies have sought to explore young people’s perceptions towards primary care pharmacy services. This study starts to provide an overview of the experiences of young people with primary care pharmacy services in England. It adds to the limited literature on these experiences and helps inform pharmaceutical services development for the future.

Limitations of the study include that the number of respondents to this survey was small. Response rates could not be determined because of the distribution methods used in this survey. A notable limitation is the potential for sampling bias, as participants were recruited from a single academic institution and were not selected using random sampling methods. This approach may have overrepresented individuals from health-related academic backgrounds, potentially influencing their awareness of healthcare services and attitudes towards pharmacists. In addition, self-selection bias is likely, as young people with a personal interest in health issues or those already living with long-term illnesses may have been more motivated to participate. This could lead to an overestimation of awareness levels, health literacy, or engagement with health services compared to the general youth population. Another important limitation is the gender imbalance among respondents, with a predominance of female participants. While this reflects the demographics of the surveyed setting, it limits the applicability of the findings to a more balanced male–female distribution. Gender differences in health-seeking behaviour, communication styles, and attitudes toward healthcare professionals are well-documented, and this imbalance could therefore skew the results. These factors combined limit the generalizability of the findings beyond the studied cohort. The results should be interpreted with caution when extrapolating to the wider population of young people in England, especially those from non-academic backgrounds, different geographical regions, or underrepresented demographic groups. Future research should aim to recruit a more diverse and representative sample to enhance external validity.

A further limitation of this study is the lack of subgroup analyses by age or condition. While the sample size and nature of the data restricted such statistical comparisons, we recognise that these analyses could have provided more nuanced insights into the perspectives of young people with differing experiences. Future research with larger and more diverse samples would allow for such analyses, thereby strengthening the evidence base. Future research should aim to address these limitations by recruiting larger and more diverse samples of young people beyond university settings, using probability-based sampling methods where feasible, and ensuring representation across a wider range of demographics and educational backgrounds.

### 4.2. Implication for Practice

Pharmacists’ interactions with young people are rarely concerned with long-term illness and its management. In contrast, primary care pharmacy services are centred on the elderly and adults [22]. This orientation may be formed from a general perception of young people’s health, i.e., that they are healthy and fit and do not often become ill. Consequently, young people are subject to being neglected by primary care pharmacy services [23].

Policymakers and primary care pharmacists in the future could utilise the perceptions and opinions of young people found in the current study to inform the development of primary care pharmacy services to meet young people’s needs and perceptions. Practical approaches include developing youth-focused, age-appropriate counselling strategies; integrating digital tools such as mobile health apps and medication reminders; and conducting targeted outreach in schools, universities, and community settings to increase awareness of pharmacists’ wider roles. Additionally, structured training and support for pharmacists are essential to enhance confidence in engaging with young people, particularly those transitioning from paediatric to adult services. While this study offers valuable preliminary insights into young people’s perceptions of pharmaceutical services provided by primary care pharmacists, only a small proportion of the data directly addressed this area, and the findings should therefore be considered exploratory rather than conclusive. Conducted within a single academic institution in England, the study nonetheless provides a useful foundation for understanding how young people engage with and perceive pharmacy-led medication services. The results may be extrapolated to similar settings and can inform the redesign of primary care pharmacy services to ensure they are inclusive of younger populations and responsive to their unique needs. Expanding this research with a larger and more diverse sample would further strengthen the evidence base and support the development of pharmacy services that better reflect the expectations, preferences, and health priorities of young people.

## 5. Conclusions

This study set out to investigate young people’s perspectives of the pharmaceutical services that are provided by primary care pharmacists relating to medication. This research found that pharmacists were not utilised at all as a source of medicine information by young people. Moreover, there is a need for further provision of pharmaceutical services and support by primary care pharmacists for young people with long-term illnesses. Previous evidence shows that this could be due to the need for further confidence when dealing with young people, the unwillingness of pharmacists to take on more responsibilities, or a shortage of training and support. To address these gaps, practical strategies are needed at both the service and system levels. Pharmacists can play a vital role in improving outcomes by offering young-centred counselling, medication adherence tools, and outreach efforts such as school-based sessions or youth-friendly pharmacy spaces. Healthcare administrators should support these efforts by promoting digital health tools tailored to young people, launching targeted educational campaigns, and integrating pharmacists into broader youth health initiatives. The results would be of benefit to the policymakers to assist in the further development of the pharmacy services. Further research could explore the impact of such interventions on young people’s engagement and health outcomes. In particular, studies may assess the effectiveness of digital tools such as medication reminder apps or SMS systems on adherence rates, or investigate young-specific barriers to pharmacist interaction through qualitative studies. These insights will further inform the design of inclusive and responsive pharmaceutical care models.

## Figures and Tables

**Table 1:** Demographic details for young people (YP) from responses to survey.

Characteristic	*n* (%)
**Gender (n = 210)**	
Male	77 (36.67)
Female	131 (62.38)
Prefer not to say	2 (0.95)
**Age range (18–24 years)**	
18	74 (35.24)
19	44 (20.95)
20	31 (14.76)
21	27 (12.86)
22	16 (7.62)
23	9 (4.29)
24	9 (4.29)
**Prevalence of long-term illness among participants**	
Diagnosed with a long-term illness	33 (15.71)
Do not have a long-term illness	177 (84.29)
**No. of long-term illness per YP (n = 33)**	
1	27 (81.82)
2	4 (12.12)
>2	2 (6.06)
**Type of Long-term illness (n = 33)**	
Skin	6
Respiratory	11
Diseases of the digestive system	5
Mental Health	3
Endocrine	7
Cardiovascular	1
Allergy Diseases of the immune system Allergic or hypersensitivity conditions	1
Other	6

**Table 2:** The relationship between participant’s age and the presence of long-term illness.

Age (Years)	Were You Diagnosed with Long-Term Illness?		Total
	Yes	No	
24	4 (12.12%)	5 (2.82%)	9 (4.29%)
23	3 (9.09%)	6 (3.39%)	9 (4.29%)
22	2 (6.06%)	14 (7.91%)	16 (7.61%)
21	5 (15.15%)	22 (12.43%)	27 (12.86%)
20	8 (24.24%)	23 (12.99%)	31 (14.76%)
19	2 (6.06%)	42 (23.73%)	44 (20.95%)
18	9 (27.27%)	65 (36.72%)	74 (35.24%)
Total	33	177	210

**Table 3:** The relationship between participants’ gender and the duration of long-term illness.

Gender	Duration of Diagnosis with Long-Term Illness								Total
	1 Year	2 Years	3 Years	4 Years	5–7 Years	8–10 Years	11–15 Years	More Than 15 Years	
**Female**	2	1	3	1	3	4	3	4	21
**Male**	0	1	3	0	2	1	0	5	12
**Total**	2	2	6	1	5	5	3	9	33

## Data Availability

The data will be made available upon request by the corresponding author. The data are not publicly available due to privacy and ethical restrictions.
